# Surgical treatment of femoral medial condyle fracture with lag screws and proximal tibial plate: A case report

**DOI:** 10.1016/j.ijscr.2020.04.060

**Published:** 2020-05-11

**Authors:** Hiroyasu Kodama, Isaku Saku, Shin Tomoyama

**Affiliations:** Department of Orthopaedic Surgery, Yaizu City Hospital, Shizuoka, Japan

**Keywords:** Case report, Femoral medial condyle fracture, Proximal tibial plate, Surgery, Knee

## Abstract

**Introduction:**

Femoral medial condyle fracture is a rare fracture. Here, we report a case of femoral medial condyle fracture treated with lag screws and proximal tibial plate as a buttress plate.

**Case presentation:**

A 80-year-old woman fell down 15 steps at her home and reported to our hospital with severe right knee pain. Radiographs and computed tomography demonstrated a femoral medial condyle fracture in the right knee (AO classification: 33-B2). Two days after injury, we performed open reduction and internal fixation using locking compression plate for proximal tibia and screws. The patient had an uneventful postoperative recovery. At the latest follow-up, the patient achieved a range of motion of 0° to 120° and could walk without pain.

**Discussion:**

Femoral medial condyle fracture is a rare fracture. As with any articular injury, anatomical restoration of the joint surface must be obtained, then lag screw fixation is required. In fractures with a vertical fracture line, a buttress plate is necessary to counteract the vertical shear forces. Nevertheless, there are no available anatomical plates that fit either the femoral medial condyle or fracture fixation, except for the relatively short plate developed for distal femoral osteotomy. We used a locking compression plate - proximal tibial plate 4.5/5.0 (Depuy Synthes TRAUMA) as a buttress plate. The implant fitted well and enhanced joint stability. Postoperative radiographs confirmed anatomical reduction, and the patient achieved a good result.

**Conclusion:**

The locking compression plate for proximal tibia is an acceptable solution for femoral medial condyle fracture.

## Highlights

• This is a case of medial femoral condyle fracture, an extremely rare fracture.
• Operation was performed with a proximal tibial plate; this is a novel case.
• The implant fitted well to the bone surface.
• This implant could be a treatment option for this fracture in other cases.

## 1 Introduction

Femoral medial condyle fracture (AO classification 33-B2) is a rare fracture [[1], [2], [3]]. To the best of our knowledge, no case reports exist of this fracture treated with a proximal tibial plate. In case of vertical fracture lines, screw fixation and buttress plates are necessary to achieve stability. However, no currently available anatomical plates fit the femoral medial condyle. Here, we present a case with femoral medial condyle fracture treated with a proximal tibial plate. This paper has been written in line with the SCARE criteria [4].

## 2 Case presentation

An 80-year-old woman was brought to our hospital with severe right knee pain after falling down 15 steps at her home. She did not present loss of consciousness, central nervous system dysfunction, or paralysis. Her vital signs were normal.

On examination, bruising and tenderness were present on her head, back, right hip, right knee, and left shoulder. The patient complained of severe pain in the right knee and could not move her knee.

**Fig. 1:**
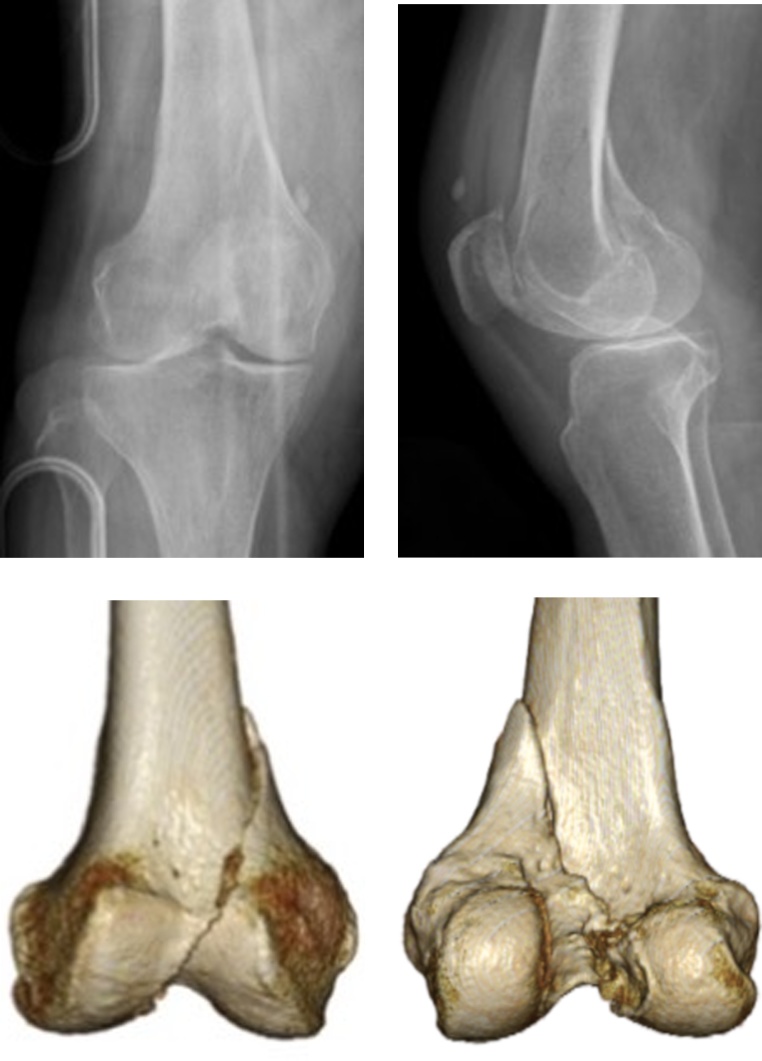
Plain radiography and computed tomography showed oblique fracture of the femoral medial condyle.

Radiography and computed tomography demonstrated a femoral medial condyle fracture of the right knee (Fig. 1). The fracture was intra-articular and simple oblique through the notch (AO classification: 33-B2.1). No intracranial hemorrhage and fracture of other sites were detected. The patient was admitted to our hospital for open reduction and internal fixation to be performed the following day. Informed consent was obtained for the surgery. Two days after the injury, we performed an open reduction and internal fixation using locking compression plate for proximal tibia and lag screws.

**Fig. 2:**
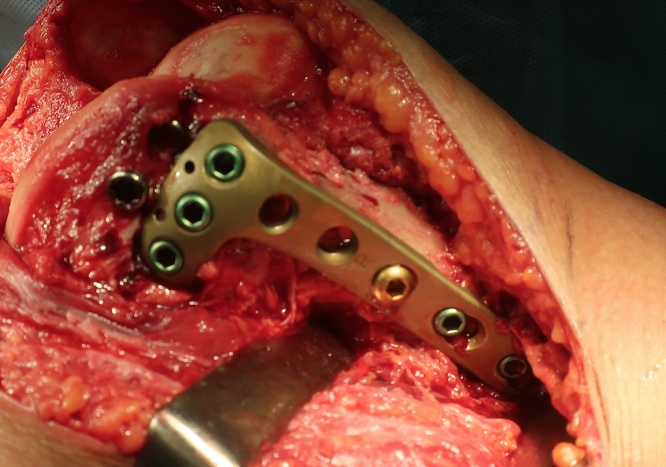
Proximal tibia plate (Depuy Synthes: LCP proximal tibial plate 4.5) was placed upside down and fixed with cortical and locking screws.

**Fig. 3:**
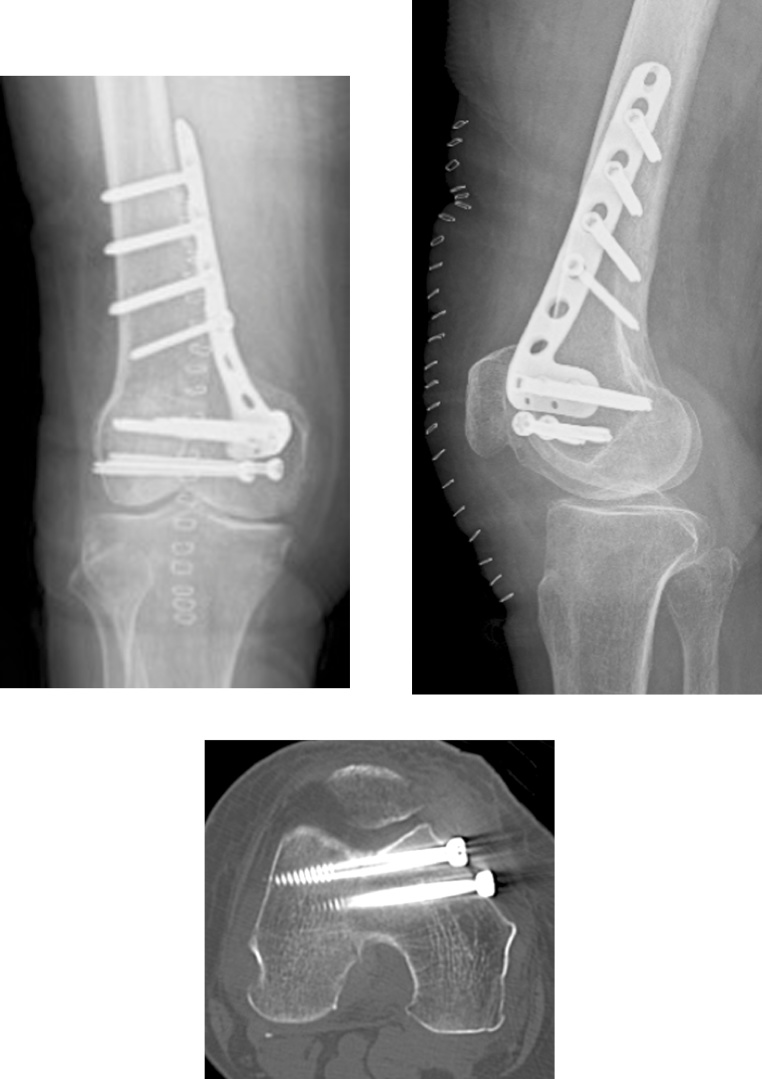
The post-operative plain radiography and computed tomography.

We gained access to the joint through the medial parapatellar approach, anatomical restoration of the joint surface was achieved with clamp application. Then, we placed the proximal tibia plate (Depuy Synthes: LCP proximal tibial plate 4.5) upside down (Fig. 2). The plate was fixed provisionally and lag screw fixation was done with two cannulated cancellous screws. The plate was bent to fit the bone surface and fixed with cortical and locking screws. Restoration, stability, and postoperative radiographs were acceptable (Fig. 3).

The patient had an uneventful postoperative recovery. Range of motion exercises and mobilized non-weight bearing were started on day one. The weight bearing protocol was: touch gait for first 4 weeks, 1/4 partial weight bearing (PWB) for weeks 4–6, 1/2 PWB for weeks 6–8, 3/4 PWB for weeks 8–10, and full weight bearing.

At the latest follow-up, the patient had a range of motion of 0° to 120° without any pain, could walk freely, and joint surface restoration was maintained radiologically.

## 3 Discussion

Isolated coronal fracture of medial femoral condyle with intact lateral femoral condyle is extremely rare [[1], [2], [3]], caused by a direct impact on the flexed knee during weight bearing [3]. Surgery is the gold standard for displaced fractures or to enable rapid return of knee function. The goal of surgical management is to promote early knee motion while restoring the articular surface, maintain limb length and alignment, and preserve the soft-tissue envelope with durable fixation that allows functional recovery during bone healing [5]. With vertical fracture lines, screw fixation alone may be insufficient, and a buttress plate should be added. However, there are no available anatomical plates that fit either the femoral medial condyle or fracture fixation, except for the relatively short plate developed for distal femoral osteotomy.

Past reports have shown the possibility of screw fixation plating for the fracture [2,[6], [7], [8], [9], [10]]. To date, however, no consensus exists regarding the optimal implant due to few cases [2]. We used lag screw fixation and plating with proximal tibial plate for the same side as a buttress plate to counteract the vertical shear forces. This is the first report on a fracture of medial femoral condyle treated with this implant. The plate fit the bone surface well, despite some bending, the clinical and radiological outcomes were good. The anatomical plate for distal medial condyle fracture of femur should be developed as soon as possible.

## 4 Conclusion

We used a proximal tibial plate upside down as a buttress plate for femoral medial condyle fracture. Although the plate needed bending to achieve congruence, it fit well and yielded a good clinical outcome. The proximal tibial plate could become the method of choice for such fractures.

## Declaration of Competing Interest

The authors declare that there is no conflict of interests regarding the publication of this paper.

## Sources of funding

Authors declare there are no funding resources for this paper.

## Ethical approval

Institutional review board approval was not required because all data were collected from clinical records and imaging systems for routine preoperative planning and follow-up.

## Consent

Written informed consent was obtained from the patient for publication of this case report and accompanying images. A copy of the written consent is available for review by the Editor-in-Chief of this journal on request.

## Author contribution

HK wrote this paper. IS attended the surgery, and all authors read this paper.

## Registration of research studies

This paper reports just the record of patient treatment. This is not a paper about research work involving human participants.

## Guarantor

Isaku Saku is the corresponding author of this paper.

## Patient perspective

The patient shared her perspective on the treatment when her wound was healed completely.

## Provenance and peer review

Editorially reviewed, not externally peer-reviewed.
